# Supplementary material for: A space hurricane over the Earth’s polar ionosphere
Source: Nat Commun. 2021 Feb 22;12:1207. doi: 10.1038/s41467-021-21459-y (PMC7900228; doi:10.1038/s41467-021-21459-y)
Supplement: Supplementary file 1 — Supplementary information [file 41467_2021_21459_MOESM1_ESM.pdf]

Supplementary Information for

A Space Hurricane over the Earth's Polar Ionosphere

Qing-He Zhang, Yong-Liang Zhang, Chi Wang, Kjellmar Oksavik, Larry R. Lyons, Michael Lockwood, Hui-Gen Yang, Bin-Bin Tang, Jøran Idar Moen, Zan-Yang Xing, Yu-Zhang Ma, Xiang-Yu Wang, Ya-Fei Ning and Li-Dong Xia

Correspondence to: zhangqinghe@sdu.edu.cn

This PDF file includes:

Supplementary Figures 1 to 2

Supplementary Figure 1

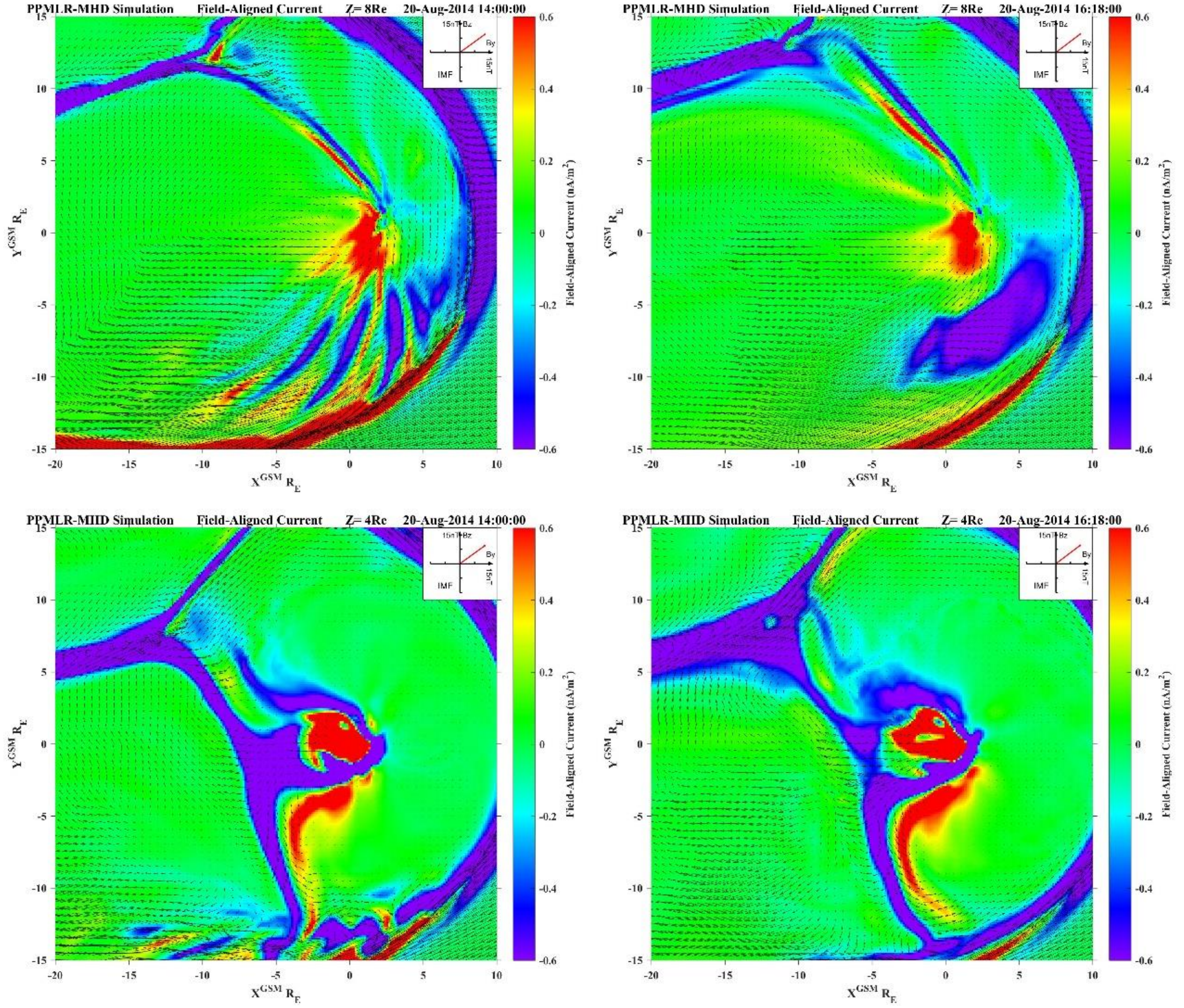

Supplementary Figure 1. Two selected snapshots of the simulated 2-D distribution of FACs and plasma velocity vectors in the X-Y plane at *Z*= 8 R_E and at *Z*= 4 R_E, respectively. The top two panels are for the simulated data in the **X-Y plane at *Z*= 8 R_E** and the bottom two are for the data in the **X-Y plane at *Z*= 4 R_E.** The two panels at left side are for the simulated data at 14:00:00 UT and the two panels at right side are for the data at 16:18:00 UT, respectively. The format of each panel is same as Figure 4**d** in the main text.

Supplementary Figure 2

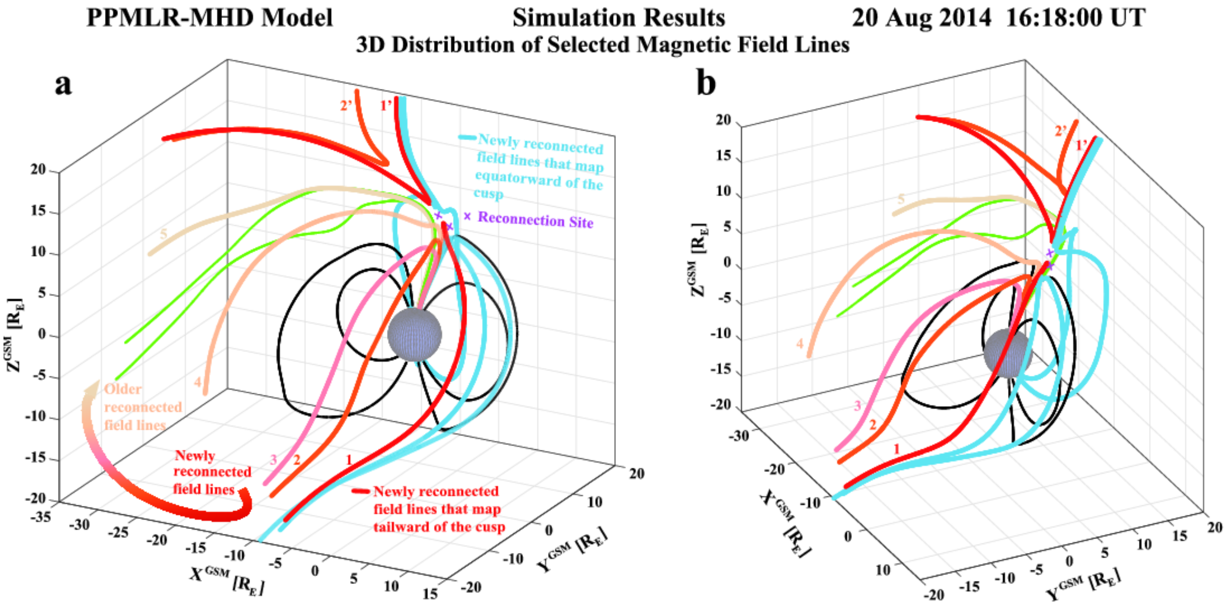

Supplementary Figure 2. Two different views of the 3-D selected magnetic field lines simulated by the PPMLR-MHD code for the snapshot shown in Figure 4. The magenta crosses represent the reconnection sites, and the numbered field lines in red to light brown represent the newly to old evolution of the reconnected field lines that also highlighted by the thick arrowed color curve. The format is same as Figure 4**b** in the main text.
