# Supplementary material for: A space hurricane over the Earth’s polar ionosphere
Source: Nat Commun. 2021 Feb 22;12:1207. doi: 10.1038/s41467-021-21459-y (PMC7900228; doi:10.1038/s41467-021-21459-y)
Supplement: Supplementary file 3 — Description of Additional Supplementary Files [file 41467_2021_21459_MOESM3_ESM.docx]

Description of Additional Supplementary Files for

**A Space Hurricane over the Earth’s Polar Ionosphere**

Qing-He Zhang, Yong-Liang Zhang, Chi Wang, Kjellmar Oksavik, Larry R. Lyons, Michael Lockwood, Hui-Gen Yang, Bin-Bin Tang, Jøran Idar Moen, Zan-Yang Xing, Yu-Zhang Ma, Xiang-Yu Wang, Ya-Fei Ning and Li-Dong Xia

Correspondence to: zhangqinghe@sdu.edu.cn

**This PDF file includes:**

**Description of** **Supplementary Movies 1 to 4**

**File name: Supplementary Movie 1**

**Description: A movie of aurora, in-situ plasma and field-aligned currents observations in the polar region of the Northern Hemisphere.** Left is the Aurora in the LBHS band (wavelength of 140-150 nm), the measured cross-track horizontal ion flows shown in mauve drift vectors perpendicular to the orbit, and the sign of the field-aligned currents (FACs) shown in red and blue color along the satellite tracks from four different DMSP satellites. Right is the field-aligned currents calculated from the magnetic field observations by the AMPERE satellites. The format is the same as Figure 2**a** and **b** in the main text, but the magnetic latitude of the AMPERE FACs maps has been extended to 60°.

**File name: Supplementary Movie 2**

**Description: A movie of the aurora in the LBHS band observed by the SSUSI instrument on board four different DMSP satellites both in the Northern and Southern Hemispheres.** The format is the same as Figure 2**a** in the main text, but without showing the measured ion flows and FACs along the satellite tracks.

**File name: Supplementary Movie 3**

**Description: A movie of the simulated field-aligned currents (FACs) and plasma velocity vectors in the GSM X-Y plane at Z= 8 R_E_ of the magnetosphere.** The format is the same as Figure 4**d** in the main text.

**File name: Supplementary Movie 4**

**Description: A Movie of 3-D selected magnetic field lines simulated by the PPMLR-MHD code for the interval of interest with a 30 seconds time resolution.** The format is same as Figure 3**b** in the main text but shows more field lines.
