# Supplementary figures and images for: A space hurricane over the Earth’s polar ionosphere
Source: Nat Commun. 2021 Feb 22;12:1207. doi: 10.1038/s41467-021-21459-y (PMC7900228; doi:10.1038/s41467-021-21459-y)

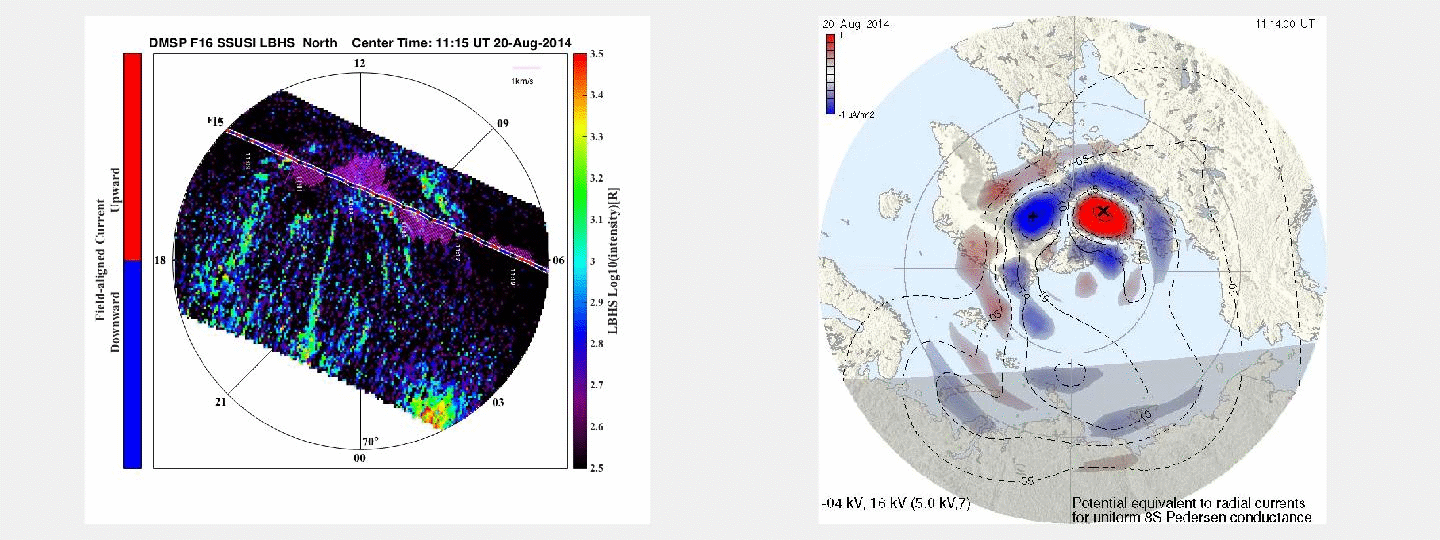

Supplement: Supplementary file 4 — Supplementary Movie 1 [file 41467_2021_21459_MOESM4_ESM.gif]

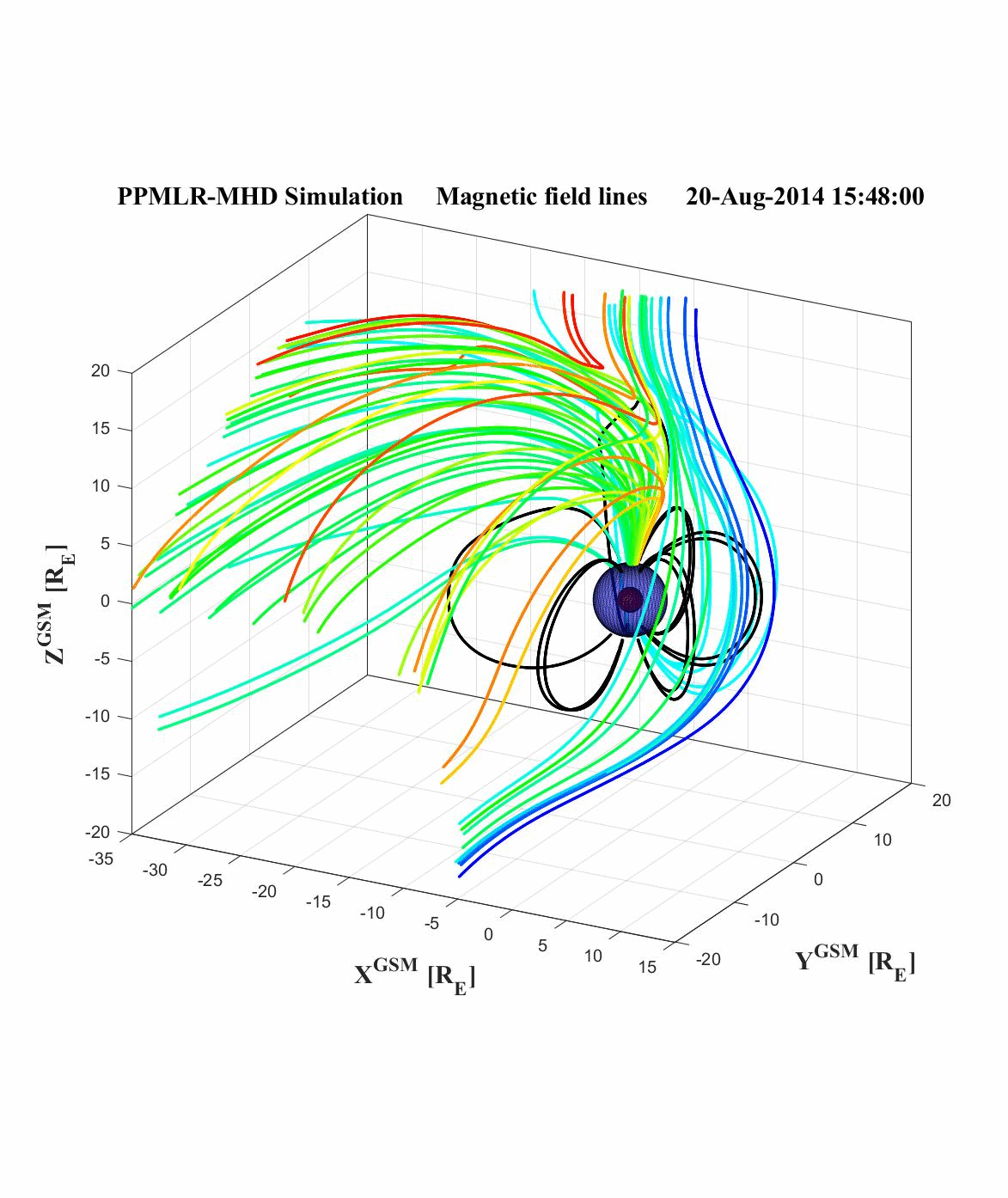

Supplement: Supplementary file 7 — Supplementary Movie 4 [file 41467_2021_21459_MOESM7_ESM.gif]
